# Self-assembly of dengue virus empty capsid-like particles in solution

**DOI:** 10.1016/j.isci.2023.106197

**Published:** 2023-02-14

**Authors:** Thais C. Neves-Martins, Nathane C. Mebus-Antunes, Carlos H.G. Neto, Glauce M. Barbosa, Fabio C.L. Almeida, Icaro P. Caruso, Andrea T. Da Poian

**Affiliations:** 1 Instituto de Bioquímica Médica Leopoldo de Meis, Universidade Federal do Rio de Janeiro, Rio de Janeiro, 21941-590 Rio de Janeiro, Brazil; 2 Centro Nacional de Biologia Estrutural e Bioimagem, Universidade Federal do Rio de Janeiro, Rio de Janeiro, 21941-590 Rio de Janeiro, Brazil; 3 Centro Multiusuário de Inovação Biomolecular e Departamento de Física, Instituto de Biociências, Letras e Ciências Exatas, Universidade Estadual de São Paulo, São José do Rio Preto, São Paulo 15054-000, Brazil

**Keywords:** Biological sciences, Molecular biology, Molecular mechanism of behavior, Virology

## Abstract

Nucleocapsid (NC) assembly is an essential step of the virus replication cycle. It ensures genome protection and transmission among hosts. Flaviviruses are human viruses for which envelope structure is well known, whereas no information on NC organization is available. Here we designed a dengue virus capsid protein (DENVC) mutant in which a highly positive spot conferred by arginine 85 in α4-helix was replaced by a cysteine residue, simultaneously removing the positive charge and restricting the intermolecular motion through the formation of a disulfide cross-link. We showed that the mutant self-assembles into capsid-like particles (CLP) in solution without nucleic acids. Using biophysical techniques, we investigated capsid assembly thermodynamics, showing that an efficient assembly is related to an increased DENVC stability due to α4/α4′ motion restriction. To our knowledge, this is the first time that flaviviruses’ empty capsid assembly is obtained in solution, revealing the R85C mutant as a powerful tool to understand the NC assembly mechanism.

## Highlights

• DENVC R85C mutant spontaneously assembles into empty capsid in solution
• Neutralization of arginine 85 in DENVC is a major trigger to capsid formation
• The disulfide cross-link between α4/α4’ increases DENVC stability favoring assembly
• R85C mutant is a powerful tool to understand the mechanism of DENV capsid assembly

## Graphical abstract

**Figure:**
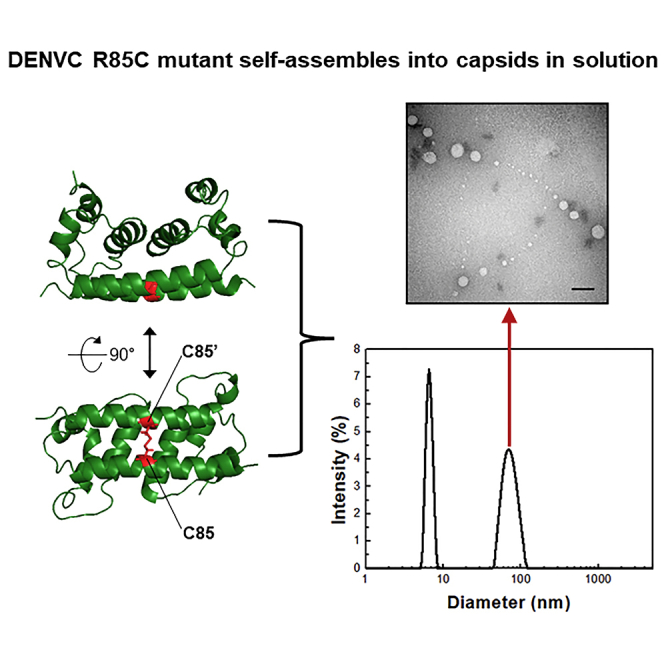

## Introduction

One of the main challenges that viruses face to be perpetuated in nature is to maintain their genome tightly protected while outside the host cell, but turn it promptly exposed after reaching the cellular environment where virus replication occurs. Capsid proteins are the viral components responsible for ensuring this task is properly performed. Capsid proteins of different icosahedral viruses contain, at their C- or N-terminal regions, positively charged arginine-rich stretches, whose net charge correlates with the size of the respective viral genome.^1^ Interestingly, the number negative charges on viral nucleic acid exceeds the number of positive charges on the capsid proteins in a ratio of order 2:1, a phenomenon known as overcharging.^2^^,^^3^ The fact that the capsid proteins’ positive charges are nonuniformly distributed, being concentrated in the protein terminal regions, which are directed toward the interior of the virion, suggests that electrostatic interactions between the capsid protein and the viral nucleic acid would be the driving force for viral NC assembly and stability. In this context, of particular interest are the flaviviruses’ capsid proteins, which, among all viruses’ capsid proteins, show the highest net charge:molecular mass ratio.^4^

The Flaviviridae family comprise important human pathogens, including dengue virus (DENV), Zika virus (ZIKV), West Nile virus (WNV), yellow fever virus (YFV), Japanese encephalitis virus (JEV) and Tick-born encephalitis virus (TBEV). DENV infection is a major public health problem worldwide, causing a broad spectrum of clinical manifestations, from mild symptoms to a severe hypovolemic shock that can be fatal, with 400 million people estimated to be infected every year.^5^ DENV is an enveloped spherical particle of 50 nm diameter. In the viral envelope, two structural proteins associate with the lipid bilayer with icosahedral symmetry, the envelope (E) and the membrane (M) proteins. Inside the shell, the viral genome, a positive-sense single-stranded RNA molecule, is packaged by multiple copies of a single protein, the capsid (C) protein.

Several cryo-EM structures have been determined for mature and immature flaviviruses, including DENV, ZIKV, WNV, JEV, TBEV, and Spondweni virus (SPOV).^6^^,^^7^^,^^8^^,^^9^^,^^10^^,^^11^^,^^12^^,^^13^^,^^14^ Although these studies provided structural details of the proteins on the virus surface, no information could be obtained for the NC structure of the mature particles, suggesting either a poorly ordered NC structure or a lack of symmetry between NC and heterotetramers formed by E and M proteins in the viral envelope.^13^^,^^14^^,^^15^

DENVC forms homodimers in solution, with each polypeptide chain containing an N-terminal intrinsically disordered region (IDR) followed by 4 α-helices connected by short loops.^16^^,^^17^ DENVC, as other flaviviruses’ C proteins, shows unique structural features, such as the predominance of quaternary contacts between α2/α2′ and α4/α4′ maintaining the dimeric structure, flexible α1 helices, and a highly electropositive surface throughout the protein,^18^ contrasting with the nonuniform charge distribution observed for most icosahedral viruses’ capsid proteins. This, together with the fact that there is no information on DENVC orientation in the capsid, makes DENVC assembly an intriguing process.

Recent work by our group showed that the *in vitro* assembly of DENV nucleocapsid-like particles (NCLPs) requires a coordinated neutralization of C protein positive charges through interaction with either size-specific nucleic acids or negatively charged surfaces.^19^ Based on structural analysis of the electropositive DENVC surface, we hypothesized that a highly positive spot conferred by arginine 85 (R85) and lysine 86 (K86) residues in DENVC α4-helix would be the first point neutralized by RNA binding, triggering NCLP assembly.

**Figure 1:**
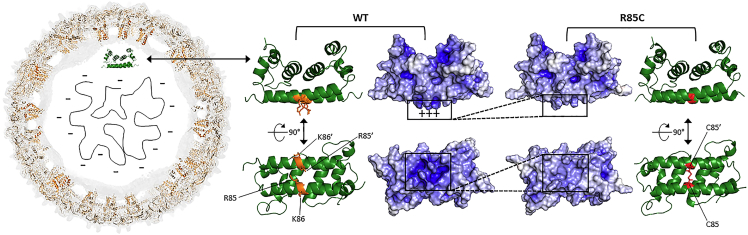
DENVC R85C mutant and the rationale of capsid *in vitro* assembly hypothesis (*Left*) Proposed orientation of DENVC in the viral structure: external density map (light gray) of the cryo-EM structure of mature DENV2 (PDB ID: 3J27), highlighting the position of E (beige) and M (orange) proteins. DENVC (green; PDB ID: 1R6R) is represented in its hypothetical position in the virion. (*Right*) Ribbon structure and electrostatic potential surface of DENVC wild-type (WT) and R85C mutant proteins in two different orientations. The structures were generated with PyMol using mutation insertion in PDB ID 1R6R, with red sticks highlighting the disulfide bond. The electrostatic potential values were calculated in APBS software with the protonation states and charge values determined by the PDB2PQR server along with the PROPKA program (pH 7.0, 200 mM NaCl, 25°C). The electrostatic potential ranges from −10 (red) to +10 kT (blue). Note that the highly positive spot in α4/α4′ is vanished in R85C mutant.

To validate this hypothesis, we designed a mutant in which DENVC R85 was replaced by a cysteine residue (R85C mutant). The rationale behind this mutation is that it would vanish the α4/α4′ positive spot (Figure 1), as well as restrict the α4/α4′ intermolecular motion due to the formation of a disulfide cross-link between α4 and α4’. Here we show by dynamic light scattering and transmission electron microscopy imaging that DENVC R85C self-assembles into capsid-like particles (CLPs) in solution, in the absence of nucleic acids, corroborating our hypothesis. Additionally, to gain insights into the thermodynamics of the DENVC/CLP dissociation/denaturation process, we performed differential scanning calorimetry, circular dichroism, and fluorescence spectroscopy experiments. We found that limiting α4/α4′motion increases DENVC stability, which could be associated with a more efficient capsid assembly. In summary, this is the first study establishing an efficient *in vitro* self-assembly of DENVC in solution in the absence of nucleic acids, revealing the R85C mutant as a powerful tool to understand the DENV NC assembly mechanism.

## Results

### DENVC R85C mutant

**Figure 2:**
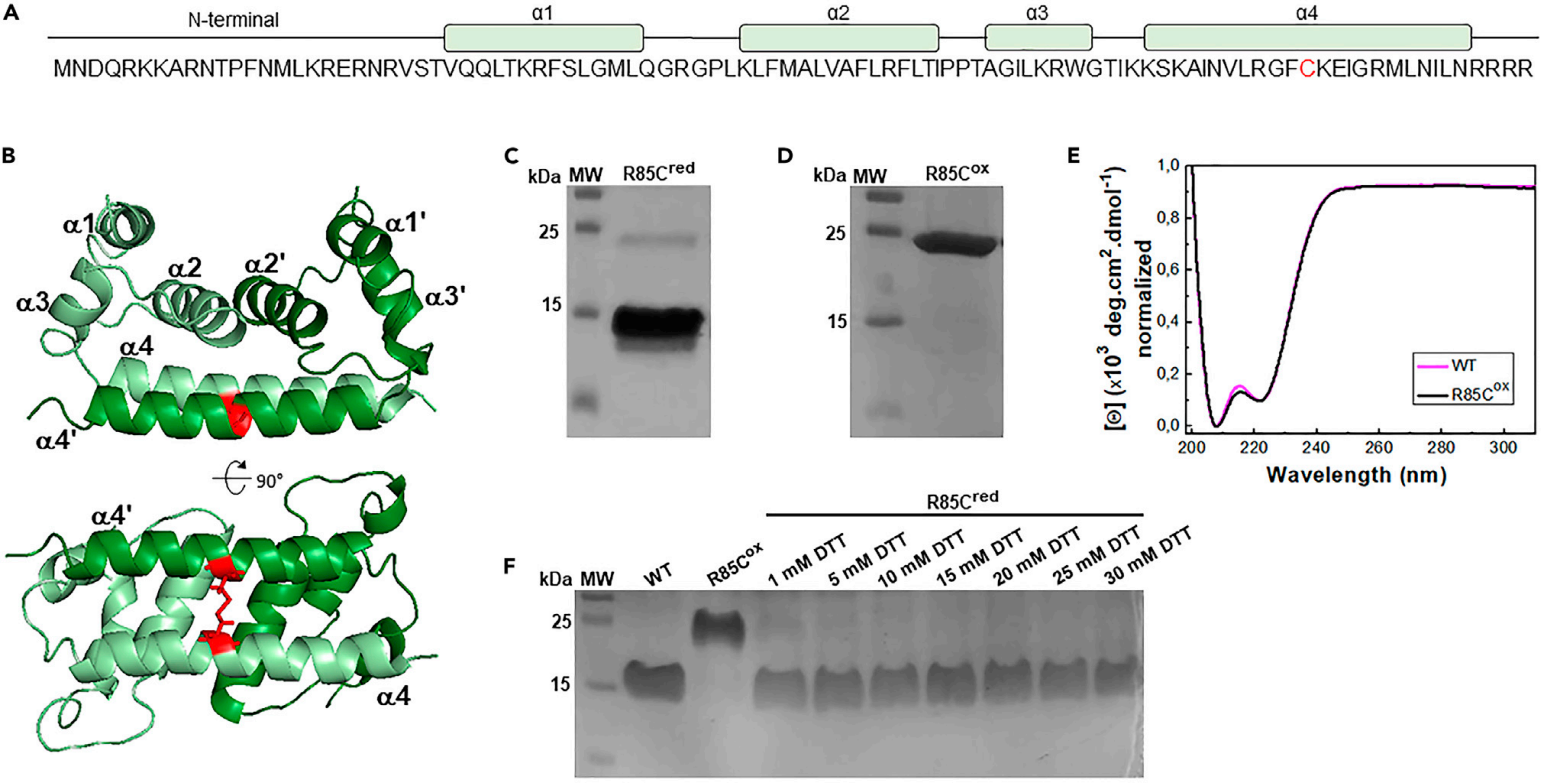
Recombinant DENVC R85C mutant in the oxidized (R85C^ox^) and reduced (R85C^red^) forms (A) Amino acid sequence (residues 1–100) of R85C mutant highlighting the cysteine residue at position 85 (red). The four helices are indicated in boxes at the top. (B) R85C ribbon structure is shown in two orientations, with the cysteines colored in red, and the disulfide bond shown with sticks. The structure was generated with PyMol introducing the mutation in PDB ID 1R6R. The monomers are differentiated into light and dark green. (C) Representative non-reduction SDS-PAGE of the sample after the first step of purification. The ∼12 kDa band corresponds to the monomer, indicating that most of the sample consists of a non-covalent R85C dimer. The ∼25 kDa band is compatible with the dimeric form of the protein, corresponding to a R85C dimer linked through a disulfide bond. MW - molecular weight standards. (D) Representative non-reduction SDS-PAGE of the sample after the second purification step showing a single band of ∼25 kDa, corresponding to the covalent dimer R85C^ox^. (E) CD spectra of WT (magenta line) and R85C^ox^ (black line), at room temperature. (F) Determination of DTT concentration necessary for the complete reduction of R85C^ox^. Representative non-reduction SDS-PAGE of the samples after incubation with the indicated concentrations of DTT for 40 min.

R85C is a DENVC mutant in which the arginine at position 85 in α4-helix was replaced by a cysteine residue (Figure 2A), removing the highly positive spot in the center region of the α4/α4′ helices’ (see Figure 1). Additionally, the mutation allows the introduction of an interchain disulfide bond, linking covalently DENVC monomers (Figure 2B) and restricting the α4/α4′ helices’ motion. To ensure the formation of the correct disulfide bond in the mutant protein, two steps during the protein purification protocol were necessary: (i) obtaining the purified protein as a non-covalent dimer by using the reducing agent dithiothreitol (DTT) in all steps of the purification protocol; and (ii) oxidizing the reduced protein with diamide to promote the disulfide bond formation (R85C^ox^). The analysis of the protein sample obtained after the first purification step in a non-reducing SDS-PAGE showed the major presence of the reduced R85C (∼12 kDa) and traces of the oxidized protein, which runs with the molecular mass of the dimer (∼25 kDa) (Figure 2C). After incubation with 3 mM diamide, only the band corresponding to R85C^ox^ was observed, indicating that all the protein was converted into covalently linked dimers (Figure 2D). To ensure the efficiency of the covalent dimer formation, we incubated R85C^ox^ with DTNB to evaluate the presence of remaining free thiol groups by the appearance of TNB^−2^ signal at 412 nm.^20^ The absence of a 412 nm band confirmed the complete formation of the disulfide bonds (data not shown). The introduction of the disulfide bond did not alter DENVC secondary structure profile, as shown by the comparison of the CD spectra of WT and R85C proteins (Figure 2E). To return the protein to its reduced form, we titrated R85C^ox^ with DTT, establishing that the incubation of the protein with 15 mM DTT is the best condition to obtain the fully reduced R85C (R85C^red^; Figure 2F).

### R85C assembles in large oligomeric particles in solution

**Figure 3:**
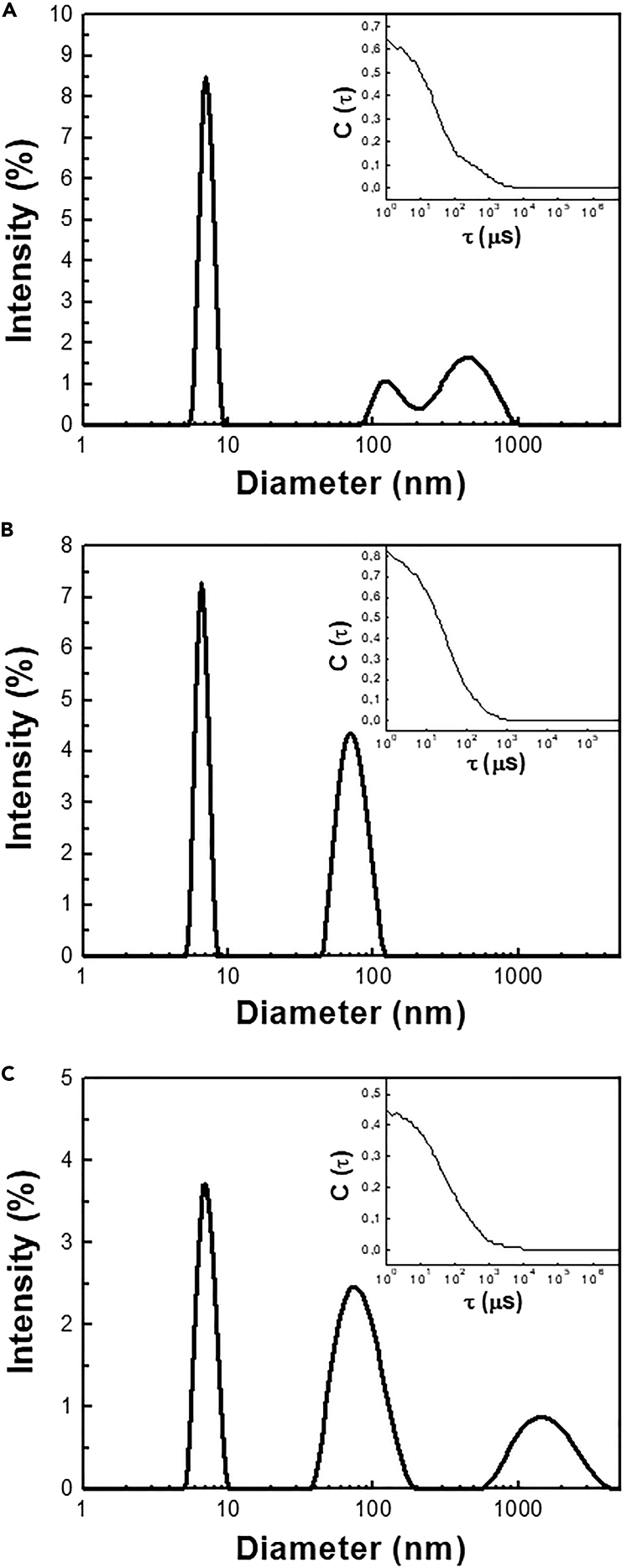
Hydrodynamic diameter distribution of DENVC WT and R85C mutant in oxidized and reduced forms in solution (A–C) Size distribution profiles of (A) WT; (B) R85C^ox^; and (C) R85C^red^ proteins. The insets show the respective correlation curves. DLS measurements were performed in a Zetasizer Nano Series S90 (Malvern Instruments) equipment, using proteins at a 200 μM concentration in 55 mM NaH_2_PO_4_ buffer (pH 7.4) containing 200 mM NaCl. The results are represented as average values from 30 scans by experiment.

To evaluate whether R85C mutant spontaneously self-assembles into capsids, we performed DLS experiments (Figure 3). The size distribution profile obtained for DENVC WT, R85C^ox,^ and R85C^red^ showed a well-defined population with apparent hydrodynamic diameters of 7.0 ± 0.1 nm, 6.4 ± 0.2 nm, and 7.0 ± 0.1 nm, respectively. The slightly more compact size observed for R85C^ox^ could be explained by the motion restriction caused by the disulfide bond. Theoretical calculation of DENVC hydrodynamic diameter, using the software HYDROPRO^21^ and considering a spherical shape for the protein, gives a value of 5.1 nm. The difference of ∼ 1-2 nm between the experimental and the calculated values may be explained by the high protein concentration used in the experiments (200 μM). At this condition, the light scattered by one particle would itself be scattered by adjacent particles, increasing the sample apparent size, a phenomenon known as multiple scattering.^22^ The high protein concentration also increases the probability of transient particle-particle interactions, affecting the sample diffusion coefficient and resulting in an increased particle apparent size. Additionally, the contribution of the high mobility of the intrinsically disordered N-terminal tails may not be ruled out.

For the WT protein, in addition to the dimer, we also observed a poorly defined population with hydrodynamic diameters ranging from ∼100 to ∼1500 nm, suggesting the presence of high molecular weight aggregates (Figure 3A). In contrast, for R85C^ox^, a second well-defined population with an apparent hydrodynamic diameter of 60 ± 2 nm was observed (Figure 3B). Different from the heterogeneous aggregates observed for the WT protein, the distribution profile of this population was very reproducible, supporting our hypothesis that the mutant self-assembles in regular structures *in vitro*. A hydrodynamic diameter of ∼60 nm is larger than that expected for the viral NC (although flaviviruses’ NC could never be observed, the viral internal area is ∼33 nm; see Figure S1). However, considering that these experiments were performed in high protein concentration and that the multiple scattering phenomena is expected to be stronger for larger particles, it is plausible to hypothesize that R85C mutant forms CLPs in solution. For R85C^red^, the second population (hydrodynamic diameter of 80 ± 3 nm) suggests that similar oligomeric structures are formed by the reduced proteins (Figure 3C). Additionally, a third population with a hydrodynamic diameter of 1459 ± 169 nm was also seen. It is interesting to note that the formation of R85C^red^ capsids suggests that neutralizing the R85 charge is sufficient to drive capsid self-assembly.

To further investigate the conditions necessary for R85C assembly, we performed the DLS analysis of protein samples after each of the two chromatography steps of the purification protocol (Figure S2). In the first step, *Escherichia coli* lysate is applied in a HiTrap Heparin HP column and the protein is purified using a step gradient with increasing NaCl concentrations. R85C is eluted from the column predominantly as a non-covalent dimer (see SDS-PAGE analysis in Figure 2C). The DLS profile obtained for this sample (Figure S2A) was very similar to that of the complete oxidized sample, indicating that either oxidized or reduced proteins were self-assembled into CLPs. This agrees with the result presented in Figure 3C, which shows that when the oxidized sample is completely reduced by incubation with 15 mM DTT (as shown in Figure 2F), the CLP population in the DLS profile is similar to that observed for the oxidized sample. Thus, the proportion of oxidized or reduced dimers does not seem to be a determinant for capsid formation. It is important to note that the sample analyzed in Figure S2A contains 1.5 M NaCl, but even in this high salt concentration, CLPs are formed. To evaluate whether the fully oxidized R85C also forms CLPs in the presence of 1.5 M NaCl, we performed a DLS analysis of the sample after the second affinity chromatography step of the purification protocol. At this step, the protein has already been completely oxidized by diamide treatment, but the salt necessary for protein elution has not been removed yet from the sample (Figure S2B). The result showed that, as for the reduced protein, R85C^ox^ also self-assembles in the presence of high salt concentrations, suggesting that DENV capsid structure is not exclusively maintained by electrostatic interactions.

### R85C self-assembles into CLPs

**Figure 4:**
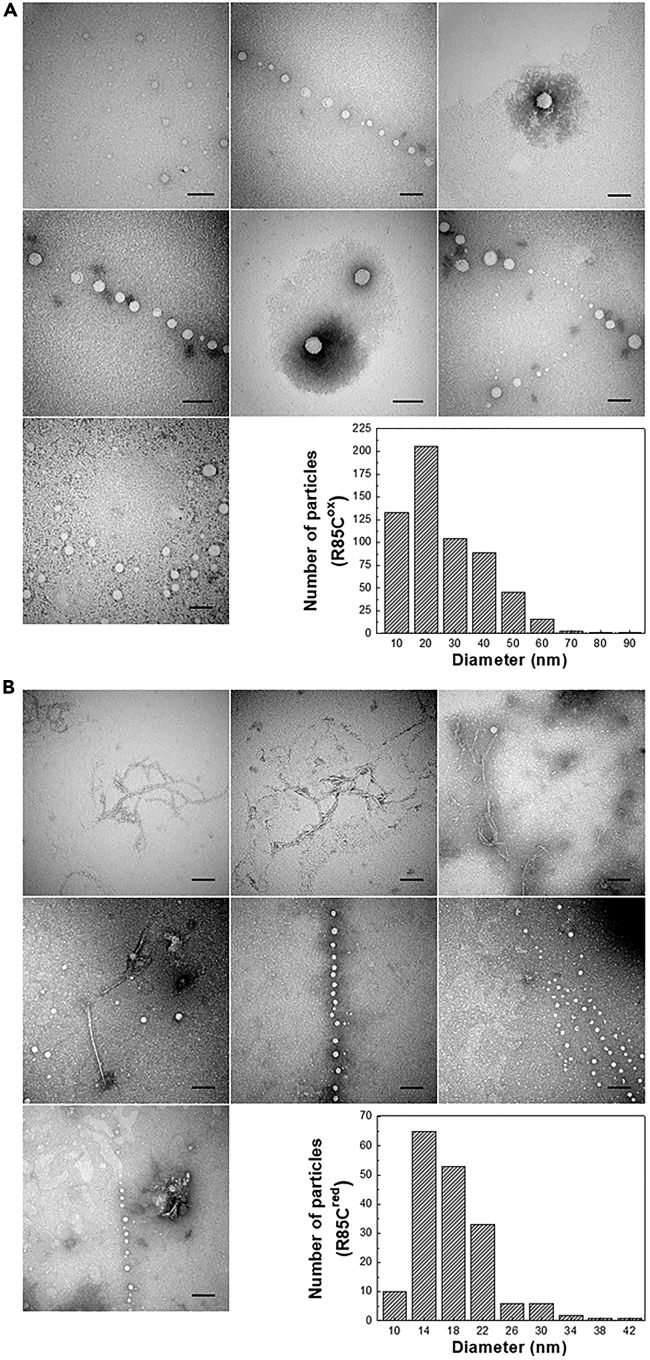
TEM images of self-assembled R85C *In vitro* assembly reaction was performed by keeping the proteins at 5 nM concentration in 55 mM NaH_2_PO_4_ buffer (pH 7.4), 300 mM NaCl, and 5 mM EDTA, for 1 h, at room temperature. Then, samples were applied to the grids, washed, and stained with 0.5% PTA. (A) Representative images of R85C^ox^ and frequency distribution of particle diameters R85C^ox^ capsids. The average diameter of particles (n = 598) was 26 ± 14 nm. (B) Representative images of R85C^red^ and frequency distribution of particle diameters R85C^red^ capsids. The average diameter of particles (n = 177) was 18 ± 5 nm. Shown are representative images from independent experiments, and all scale bars represent 100 nm. Particles’ counting and Feret’s diameter measurements were performed using ImageJ software.

To obtain additional information on R85C self-assembled oligomers, we carried out TEM imaging experiments. For R85C^ox^ samples, we observed well-defined structures, compatible with the size and shape expected for DENV NC, with an average Feret’s diameter of 26 ± 14 nm (Figure 4A). This result further supports our hypothesis that the neutralization of R85 positive charge in the DENVC due to RNA binding triggers DENVC NC assembly. It is noteworthy that, in contrast to the DLS size distribution profiles, particles’ size histograms obtained in the analysis of TEM images did not result in normal distributions. The DLS data likely reflect the behavior of an ensemble in solution, for which a Gaussian distribution is expected. On the other hand, since for TEM, the particles are adsorbed on the grid surface, the observed image is a sample snapshot. However, despite these differences, the particle sizes sampled by both methods are of the same magnitude and comparable, considering that DLS size measurements are affected by the high protein concentration used in the experiments, which favors the multiple scattering and transient particle interaction (see previous section).

R85C^red^ also formed CLPs, but the observed particles were on average smaller than those seen for R85C^ox^, with an average Feret’s diameter of 18 ± 5 nm (Figure 4B). This behavior is different from that observed in DLS experiments, in which slightly larger diameters were found for R85C^red^ particles (Figures 3B and 3C). These differences would be explained by the surface properties of the particles generated by oxidized or reduced R85C mutant, which would influence particle adsorption on the grids. Although this is an interesting hypothesis, further experiments are necessary to conclusively explain these results. Additionally, differently from the observed for R85C^ox^, heterogeneous filamentous structures were also seen for the reduced protein (Figure 4B). It is known that microscopy techniques favor surface-driven oligomerization, which indeed occurs with DENVC.^19^ Since DLS results indicate that these filaments occur in negligible amounts in solution, we would speculate that surface-driven assembly of filament-type structures would be favored for the reduced protein. Whether these structures play a role in the virus life cycle requires further investigation.

### Thermodynamics and structural stability of DENVC WT and R85C mutant

The R85C mutant was designed both to remove the positively charged site and to restrict the intermolecular motion in DENVC α4/α4′ helices. DLS and TEM results strongly support that the neutralization of R85 is crucial for capsid assembly since structures compatible in size and shape with DENV capsids were formed either with R85C^ox^ or with R85C^red^. Nonetheless, when the cysteine residues are not covalently linked (R85C^red^), assembly seems to be less efficient, with the formation of filamentous structures, possibly due to less coordinated protein interactions caused by α4/α4′ helices’ dynamics. Thus, to investigate the effects of restricting α4/α4′ helices motion on DENVC oligomerization reaction, we performed thermal and chemical denaturation experiments aiming to characterize the thermodynamics of the dissociation/denaturation processes of DENVC WT, R85C^ox^, and R85C^red^, and the respective CLPs.

### Thermal denaturation studies

**Figure 5:**
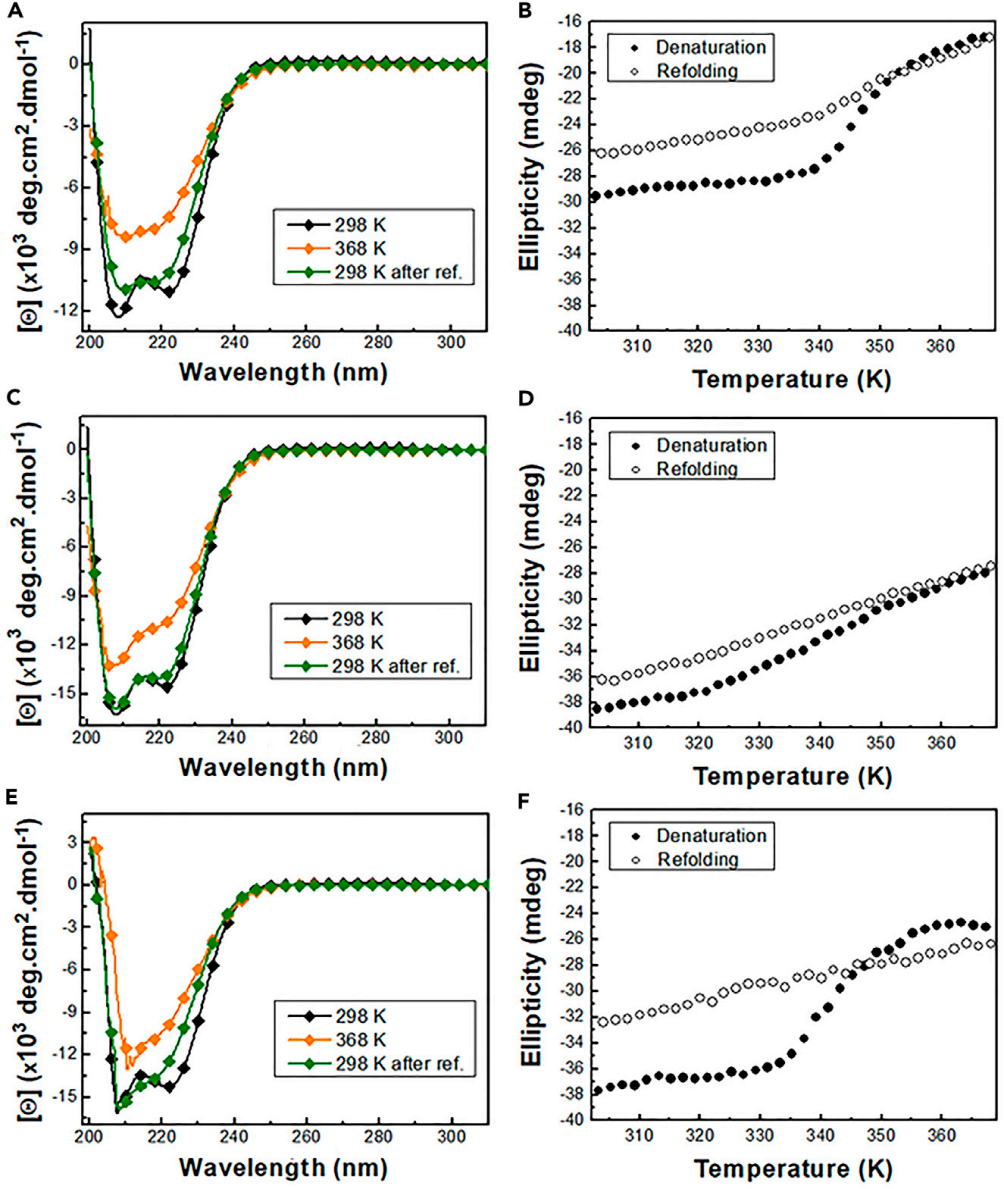
CD analysis of DENVC WT, R85C^ox^, and R85C^red^ thermal dissociation/denaturation process (A, C, E) CD spectra of WT (A), R85C^ox^ (C), and R85C^red^ (E), at 298 K (black symbols), 368 K (orange symbols), and after return to 298 K (green symbols). (B, D, F) Thermal dissociation/denaturation curves of WT (B), R85C^ox^ (D), and R85C^red^ (F) proteins were obtained by collecting the ellipticity at 222 nm from 298 to 368 K (filled circles) and return (empty circles). The data were obtained with a scan rate of 1.0 °C/min. The samples were prepared at 13 μM concentration in 55 mM NaH_2_PO_4_ buffer (pH 7,4) with 200 mM NaCl, and all experiments were performed at least twice.

To analyze the structural stability of DENVC WT and mutant proteins, we used circular dichroism spectroscopy (CD) to compare their thermal dissociation/denaturation processes (Figure 5). For the interpretation of the results, it is important to bear in mind that the flaviviruses’ C proteins form intertwined homodimers stabilized predominantly by quaternary contacts, making it very unlike the existence of a folded monomer in solution.^18^ Thus, the DENVC dimer dissociation process cannot be analyzed separately from monomer denaturation, so we should interpret the data as resulting from the transition from a folded dimer to an unfolded/disordered monomer or dimer.

As expected, at room temperature, WT, R85C^ox^, and R85C^red^ showed a typical CD spectral pattern of α-helical proteins, with negative bands at 208 and 222 nm (Figures 5A, 5C, and 5E, respectively). Thermal dissociation/denaturation profiles for each protein were analyzed by following the decrease in ellipticity at 222 nm (i.e., decrease in α-helix content) from 298 to 368 K (25-95°C). The denaturation curves obtained for WT and R85C^red^ proteins were very similar, reaching a disordered state with remaining residual α-helix content, as observed at 368 K (Figures 5A and 5E). Additionally, the curves showed a sigmoidal profile typical of a cooperative process (Figures 5B and 5F - filled circles). On the order hand, for the R85C^ox^, a non-cooperative process (Figure 5D) that did not reach the same degree of disorder as observed for DENVC-WT and the R85C^red^ (Figure 5C) was observed, indicating that the immobilization of α4/α4′ helices stabilize DENVC. We decided not to calculate the thermodynamic parameters from these data for the following reasons: (i) complete protein unfolding was not reached, (ii) the unfolding process was not completely reversible, and (iii) the samples did not present the same initial conformational states (dimer for WT and a mixture of dimer and CLPs for the R85C). Although we did not observe a complete refolding for any of the proteins, the reversibility was improved upon dilution (Figure S3).

**Figure 6:**
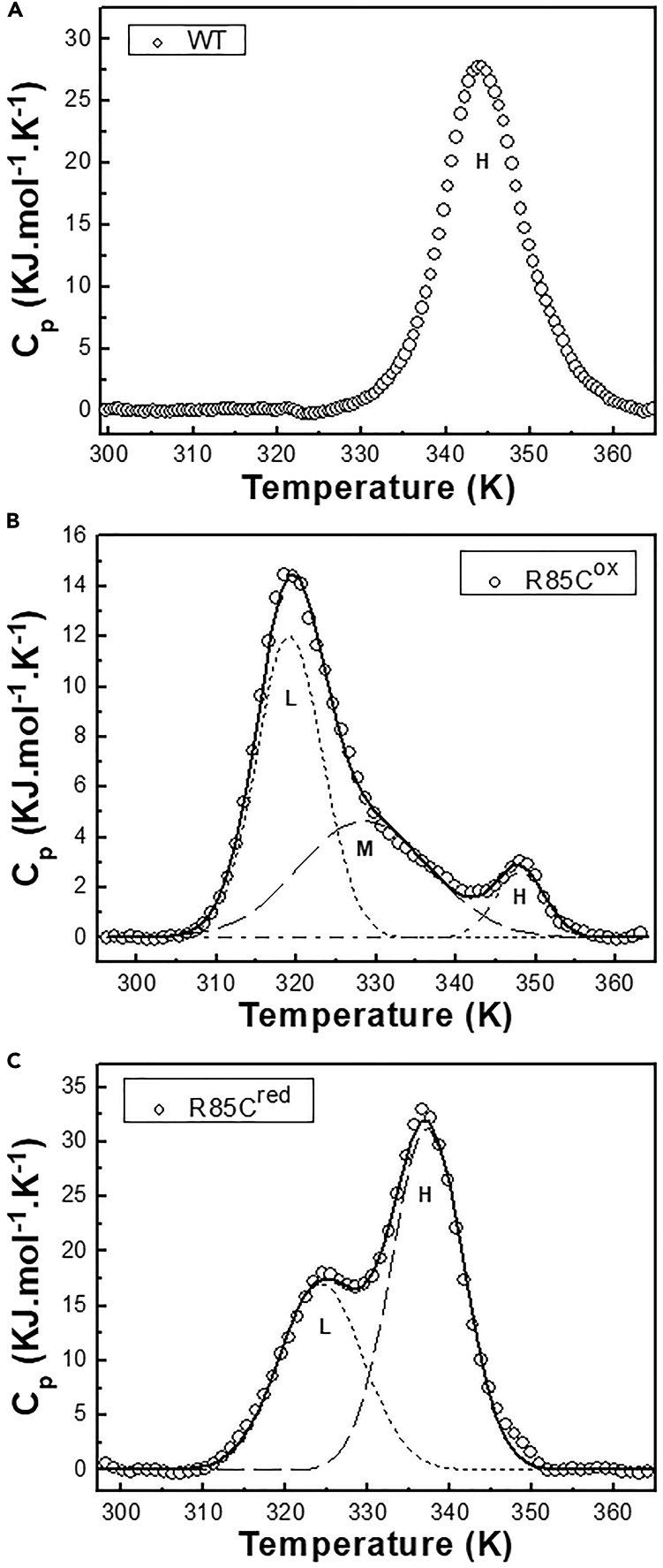
DSC analysis of DENVC WT and R85C mutant thermal dissociation/denaturation processes (A–C) DSC thermogram of DENVC WT (A), R85C^ox^ (B), and R85C^red^ (C), with the Gaussian fit for each transition shown by the dashed lines. The baseline was adjusted using Launch NanoAnalyze software. The samples were prepared at a 42.5 μM concentration in 55 mM NaH_2_PO_4_ buffer (pH 7.4) with 200 mM NaCl, and measurements were performed from 293 to 368 K with a scan rate of 1°C/min.

To gain more insight into the energetics of the dissociation/denaturation processes of DENVC WT and mutant proteins, we performed DSC experiments. The thermograms obtained for DENVC WT, R85C^ox^, and R85C^red^ are shown in Figures 6A, 6B, and 6C, respectively. Thermogram deconvolution of R85C mutant was carried out to generate the Gaussian fits from which the $T_{m}$, the calorimetric enthalpy variation (${\Delta H}_{cal}$), and the Van’t Hoff enthalpy variation (${\Delta H}_{VH})$ were obtained for each transition (Figure S4).

The thermogram obtained for WT protein showed a single transition (Figure 6A), which can be interpreted as the denaturation of the folded dimer (D) to a monomer (M). The corresponding transition for R85C^ox^, which in fact is a transition from a folded dimer to a disordered dimer ($D^{d})$ due to the covalent bond linking the monomers, showed a slightly higher $T_{m}$ when compared to that of the WT protein (347.9 ± 0.1 K for R85C^ox^ and 344.1 ± 0.2 K for WT; Table 1), corroborating the increased stability conferred by the disulfide bond (Figure 6B). Curiously, for R85C^red^ this transition occurred at a lower temperature ( $T_{m}$ = 337.19 ± 0.01 K), which would be explained by a direct effect of the R85 substitution on protein stability (Figure 6C). The ${\Delta H}_{VH}/{\Delta H}_{cal}$ of ∼1 for WT and R85C^red^ proteins (Table 1) indicates that the transition from (D) to (M) obeys a two-state equilibrium, which is not valid for R85C^ox^.

**Table 1:** Thermodynamic parameters for the thermal dissociation/denaturation of DENVC WT, R85C^ox^, and R85C^red^

Protein	Transition^a^	Interpretation	$T_{m}$ (K)	${\Delta H}_{cal}$ (kJ.mol^−1^)	${\Delta H}_{VH}$ (kJ.mol^−1^)	$\frac{{\Delta H}_{VH}}{{\Delta H}_{cal}}$
WT	H	D⇌M	344.1 ± 0.2	329 ± 8	326 ± 4	0.99 ± 0.5
R85C^ox^	L	CLP⇌Un	319.2 ± 0.1	121 ± 6	354 ± 3	2.9 ± 0.5
	M	Un⇌D	328.2 ± 0.4	86 ± 12	192 ± 11	2.2 ± 0.9
	H	$D⇌D^{d}$	347.9 ± 0.1	17 ± 6	633 ± 131	37.2 ± 21.8
R85C^red^	L	CLP⇌D	325.0 ± 0.9	221 ± 4	294 ± 4	1.3 ± 1
	H	D⇌M	337.19 ± 0.01	349 ± 7	362 ± 3	1.04 ± 0.4

a H, L, M stand for transitions at high, low, and medium temperature ranges, respectively; and *CLP*, *D*, *Un*, *D^d^*, and *M* stand for capsid-like particle, dimer, unknown state, disordered dimer, and monomer, respectively.

Consistent with DLS and TEM results, which showed the formation of CLPs by the mutant proteins, R85C^ox^, and R85C^red^ thermograms presented an additional transition at lower temperatures (Figures 6B and 6C), probably corresponding to CLP dissociation to folded species since no changes in the protein structures were observed at the respective temperature range (see Figures 5D and 5F). The comparison of the $T_{m}$ of these transitions (319.2 ± 0.1 K for R85C^ox^ and 325.0 ± 0.9 K for R85C^red^) suggests that the stabilization of the dimer through the immobilization of the α4/α4′ helices is an important step in capsid assembly.

Finally, a shoulder in the R85C^ox^ thermogram indicates the occurrence of an extra conformational transition at an intermediate temperature range (Figure 6B). The helix content variation within this temperature range (from 315 to 340 K; see Figure 5D) suggests that the protein is still folded, so we represented this transition as the equilibrium between two folded states, an unknown state (*Un*), which could be a dimer of a capsomer that is in equilibrium with the disordered dimer (*D*^*d*^). Additionally, it is interesting to note that this extra transition is compatible with the non-cooperative denaturation process observed R85C^ox^ (see Figure 5D).

### Chemical denaturation studies

**Figure 7:**
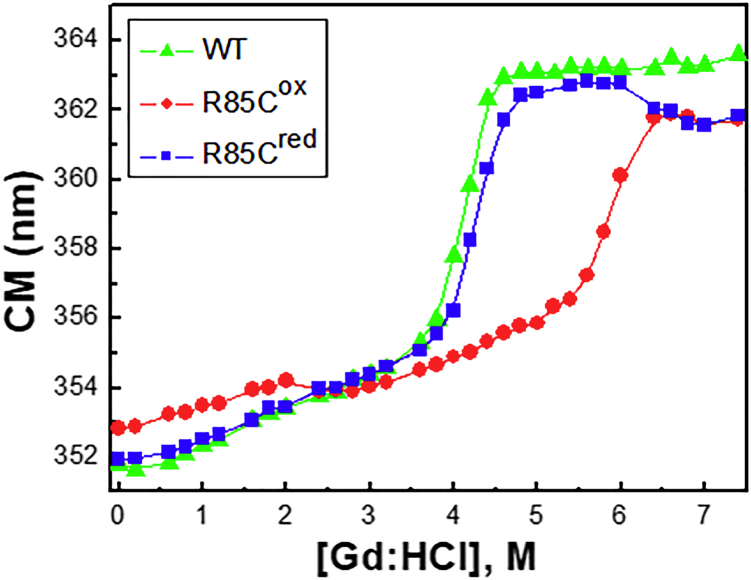
Chemical denaturation of DENVC WT, R85C^ox^, and R85C^red^ Effects of Gd:HCl on the intrinsic fluorescence spectra of WT (green triangles), R85C^ox^ (red circles), and R85C^red^ (blue squares) proteins. The samples were incubated with Gd:HCl concentrations ranging from 0 to 7.4 M in 55 mM NaH_2_PO_4_ buffer (pH 7.4), containing 200 mM NaCl. Tryptophan fluorescence spectra were run exciting the samples at 280 nm and collecting fluorescence emissions from 290 to 450 nm, at 25°C. All experiments were carried out at least twice.

Since it was not possible to completely denature R85C^ox^ using temperature, we performed a chemical denaturation assay using guanidine hydrochloride (Gd:HCl) within concentrations ranging from 0 to 7.4 M (Figure 7). Denaturation was monitored by measuring the redshift in the center of mass of proteins’ intrinsic fluorescence emission spectra. In agreement with the thermal denaturation results, WT and R85C^red^ chemical denaturation profiles were very similar, both showing sigmoidal curves, confirming the cooperative nature of the process. The Gd:HCl concentrations that resulted in 50% denaturation were 4 and 4.2 M for WT and R85C^red^, respectively. R85C^ox^ showed a different behavior, displaying two transitions, the first occurring between 0 and 3 M Gd:HCl and the second between 3 and 7.4 M. Furthermore, a considerable increase in stability was observed for the R85C^ox^, with the denaturation midpoint at 5.6 M Gd:HCl, confirming that the immobilization of α4/α4′ helices plays an important role in DENVC stabilization.

## Discussion

Flaviviruses’ NC formation is an intriguing issue. Although the steps of virus morphogenesis and maturation involving E and prM/M proteins are relatively well understood, there is no information on how the C protein recognizes and encapsidate the viral genome as well as on how NC is structured into de virion. The currently available cryo-EM structures for several flaviviruses showed details on the structural organization of E and M proteins on the virus surface.^13^^,^^14^^,^^15^^,^^23^^,^^24^^,^^25^^,^^26^ For mature particles, a smooth icosahedral outer surface is formed by 90 E protein dimers (Figures S1A and S1B). M proteins, which are mostly transmembrane, associate with the transmembrane helices of E proteins. For all mature cryo-EM structures obtained, no densities were observed between the inner membrane layer and the virus (Figures S1A and S1B). Interestingly, for immature particles of ZIKV, a residual density was observed in contact with the transmembrane domains of the E and M proteins (Figure S1C), but even in this case, it was not possible to obtain conclusive information on NC structural organization.

To contribute to the understanding of flaviviruses' NC assembly process, we designed a DENVC mutant that self-assembles into regular empty CLPs. To our knowledge, this is the first time that flaviviruses CLP assembly is obtained in solution in the absence of any nucleic acid. Our strategy was to mimic in the mutant (DENVC R85C) two possible effects of RNA binding to DENVC: specific charge neutralization and protein immobilization. This was achieved by (i) removing the positively charged spot located in the central region of the α4/α4′ helices; and (ii) restricting helices’ motion by introducing an interchain α4/α4′ covalent bond. TEM imaging showed that the average diameter of R85C CLPs is quite consistent with the internal area of the virion shown in the flaviviruses’ cryo-EM structures (Figure S1), supporting the hypothesis that they are structurally similar to the actual DENV NCs.

**Figure 8:**
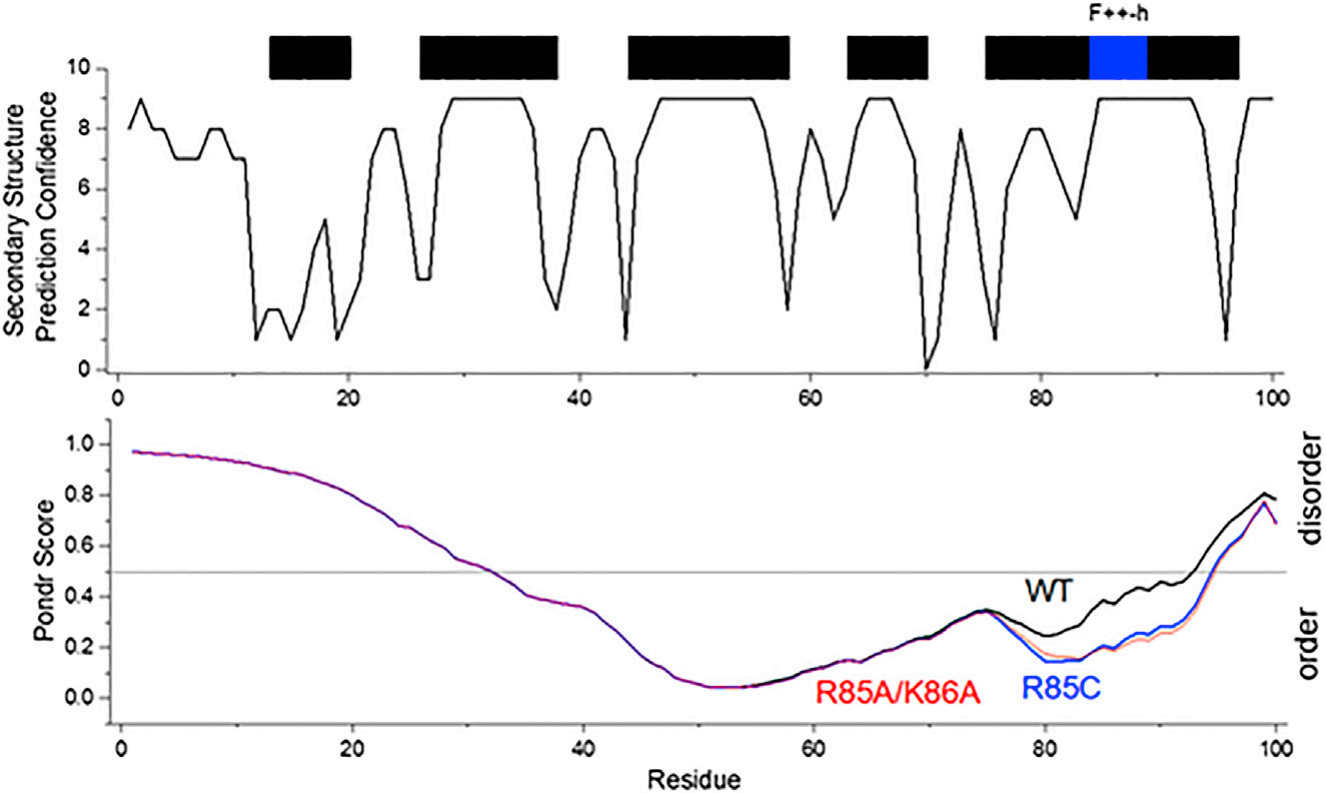
Prediction of the secondary structure and order degree of DENVC-WT and R85C and R85A/K86A mutants JPRED4 confidence level^48^ (at the top) and PONDR VSL2 order/disorder prediction^49^^,^^50^ (at lower).

The fact that both the oxidized (R85C^ox^) and reduced R85C (R85C^red^) DENVC mutants assemble into CLPs suggests that charge neutralization is the major trigger DENV NC formation. However, as shown by both DLS and TEM, R85C^red^ formed large aggregates not observed for R85C^ox^, suggesting that the motion restriction of α4/α4′ makes the capsid assembly process more efficient. Our recent observation that DENVC-WT formed empty CLPs when the protein was incubated over negatively charged surfaces^19^ reinforces the importance of restricting local protein mobility for NC assembly. Here we showed using different approaches that the immobilization of α4/α4′ helices plays an important role in DENVC dimer stabilization. Thermal denaturation experiments revealed an increased $T_{m}$ for R85C^ox^ dimer denaturation when compared to the reduced form or the WT protein (347.9 ± 0.1 K for R85C^ox^ compared to 337.19 ± 0.01 K and 344.1 ± 0.2 for R85C^red^ and WT proteins, respectively). The same was found in chemical denaturation experiments (denaturation midpoint at 5.6 M Gd:HCl for R85C^ox^ compared to 4.0 M and 4.2 M Gd:HCl for WT and R85C^red^, respectively). Altogether, these results demonstrate that DENVC α4/α4′ helices’ immobilization increases the dimer stability, which would be a role played by the viral RNA during the assembly process. However, based on theoretical studies on viral capsid assembly,^27^ we can also speculate that the interchain covalent bond would change the capsid proteins’ stiffness and thus increase the capsid stability favoring the assembly process. To further address this point, we used JPRED and PONDR VSL2 servers to simulate the tendencies for secondary structure and order/disorder along with WT and mutant DENVC sequences (Figure 8). PONDR VSL2 predictions were consistent with the known ordered regions from the structure of the dimer.^17^ Interestingly, VSL2 prediction suggested an increased tendency of disorder at α4/α4′ when compared to the α2/α2′ hydrophobic cleft, which showed the highest predicted order. Accordingly, JPRED secondary structure prediction showed lower confidences at its N-terminal portion, while the highest confidence was observed at its central region, where the conserved signature F++-h (FRKEI for DENVC-WT) is located.^18^ Remarkably, when PONDR VSL2 prediction was applied to R85C mutant, a significant increase in α4/α4′ predicted order was found. Thus, one can speculate that, during the virus replication cycle, the relative mobility in α4/α4′ would be essential for C protein interaction with the viral genome, which increases helices' order and stability, triggering NC assembly and preventing the formation of empty capsids or defective virus-like particles. The intrinsic motion of the capsid proteins may also be present in the assembled virus particle and may contribute to the absence of electron density related to NC in the cryo-EM reconstructed structures.^6^^,^^13^^,^^14^ Curiously, a PONDR VSL2 prediction behavior similar to that found for R85C was observed for the DENVC R85A/K86A double mutant previously characterized by Teoh and cols,^28^ suggesting that the removal of the α4/α4′ positively charged spot itself would be sufficient to restrict α4/α4′ mobility.

Charge neutralization is thought to be the major trigger of NC assembly of RNA viruses. In contrast to the double-stranded DNA viruses, which assemble their capsids before genome packaging, single-stranded RNA viruses generally package their genomes simultaneously to capsid assembly.^29^ Indeed, either for flaviviruses or for alphaviruses, which are also small enveloped +ssRNA viruses, attempts for reproducing NC assembly *in vitro* required capsid protein charge neutralization.^28^^,^^30^^,^^31^^,^^32^ Thus, it is expected that genomic RNA elements, namely negative charges, and long length, drive the orientation of capsid proteins to allow the protein-protein interaction necessary to build the capsid. Our TEM results showed an average diameter size of ∼30 nm for the CLPs formed by the R85C^ox^ mutant. Considering a DENVC dimer area of 15 nm^2^ (estimated from the protein structure; 1R6R), we would speculate that approximately 180 dimers are necessary to stabilize the capsid by C protein-C protein interactions.

Flaviviruses’ NC assembly seems to be a very coordinated process, making it difficult to reproduce NC formation *in vitro*, as reflected by the few examples described in the literature. For DENVC and TBEVC, NCLPs could be observed by TEM after protein incubation with single-stranded nucleic acids.^28^^,^^33^^,^^34^ The particles were more homogeneous in size and shape for larger nucleic acids, especially the full-length viral RNA, while no particles were obtained in the absence of nucleic acid. Recently, we further characterized DENVC NCLP *in vitro* assembly.^19^ We showed a stoichiometry of 1:1 (DENVC:oligonucleotide) molar ratio for 5-mer or 25-mer oligonucleotides, and that DENVC empty capsids could be formed in a surface-driven assembly process. Additionally, we hypothesized that DENV NC formation would require the neutralization of a specific positively charged spot in α4-helix (R85 and K86 residues) instead of an unspecific electrostatic effect on the entire α4/α4′ protein face.^19^ Here we confirmed this hypothesis by showing that the removal of the positive charge at position 85 in R85C DENVC mutant is to be the major trigger driving to empty capsid assembly since the reduced protein (R85C^red^) also assembled as empty capsids. Interestingly, a DENV infectious clone expressing the DENVC double mutant R85A/K86A produced a functional C protein and generated infectious virus particles, although with decreased infectivity.^28^ This supports the hypothesis that the removal of the positively charged spot in DENVC α4/α4′ helices doesn’t affect the overall protein structure but increases the probability of the generation of viral defective particles.

The assembly process of alphaviruses’ NC is much better understood than that of flaviviruses. In contrast to the observed for the flaviviruses, cryo-EM structures of the alphaviruses clearly showed icosahedral capsids in which the C-terminal chymotrypsin-like domain of the capsid proteins (known as core protein, CP) interacts with neighboring CPs and with the envelope E2 proteins.^35^^,^^36^^,^^37^^,^^38^^,^^39^^,^^40^^,^^41^^,^^42^^,^^43^^,^^44^ The disordered CP N-terminal positively charged domain (about 120 residues, for which ∼30 are positively charged) is thought to interact with the viral genome in the virion core.^45^ Alphaviruses’ CP assembles into core-like particles (CLPs) *in vitro* through the interaction with different polyanions, such as single-stranded DNA or RNA oligonucleotides of at least 14 bases in length, heparan sulfate, or PEG-coated nanoparticles.^46^ Remarkably, mutations in Ross River Virus (RRV) CP disordered N-terminal domain that replaced 4 lysine residues (K104-107) by aspartic residues (4D mutant) led CP to assemble in empty capsids,^31^ confirming that CP N-terminal charge neutralization is the trigger for NC assembly. Additionally, when an anionic RRV CP mutant, in which all the N-terminal positively charged residues were replaced by negatively charged ones, was mixed with the WT protein, they assembled into CLPs,^32^ further supporting the CP N-terminal charge neutralization mechanism for alphaviruses NC assembly.

Like alphaviruses’ CP, flaviviruses’ C proteins also contain a disordered positively charged N-terminal region. Nevertheless, this region has never been considered the main site for viral RNA interaction during NC assembly. This is possibly a consequence of the excessive emphasis given to the asymmetric charge distribution model for flaviviruses C protein, which considers that the positive charges are concentrated in the α4 helix despite the higher proportion of positively charged residues in the N-terminal region (8 of 24 residues). Interestingly, a TBEVC mutant truncated at the N-terminal end (TBEV-Δ16-C) did not form NCLPs under the same conditions that other flaviviruses’ C proteins, such as full-length DENVC, assemble.^47^ Thus, although not considered so far, a different orientation of flaviviruses’ C proteins in the virion in which the N-terminal region interacts with the viral RNA in the central core cannot be ruled out.

### Limitations of the study

We were successful in determining the formation of empty CLPs, but we do not have information on the dimer/CLP equilibrium so far. DLS informed on the coexistence of CLP and dimer of R85C^ox^ but this technique does not allow to determine the concentrations of each species. Thus, further studies are necessary to quantify the CLP/dimer equilibrium for the mutant without nucleic acid and the wild-type DENVC in the presence of nucleic acids,^19^ which will contribute to the understanding of capsid dynamics in the context of the virus particle.

## STAR★Methods

### Key resources table

**Table:** 

REAGENT or RESOURCE	SOURCE	IDENTIFIER
**Bacterial and virus strains**		

*Escherichia coli* BL21-DE3-pLysS	Thermo Fisher Scientific	C606010

**Chemicals, peptides, and recombinant proteins**		

Diamide	Sigma-Aldrich	Cat#D3648
Dithiothreitol (DTT)	Sigma-Aldrich	Cat#D0632
Isopropyl β-D-1-thiogalactopyranoside (IPTG)	Sigma-Aldrich	Cat#I5502
Phosphotungstic acid (PTA)	Sigma-Aldrich	Cat#79690
Guanidine hydrochloride (Gd:HCl)	Sigma-Aldrich	Cat#G4505
Protease Inhibitor Cocktail	Sigma-Aldrich	Cat#P8465
DENVC R85C mutant	GenScript	N/A

**Deposited data**		

Raw data	This paper; Mendeley Data	https://data.mendeley.com/datasets/prx938vv7h/draft?a=d02e7cac-67a8-4963-815f-b5e4c6bd33b9; https://doi.org/10.17632/prx938vv7h.1

**Software and algorithms**		

JPRED4	Drozdetskiy et al., 2015^48^	https://www.compbio.dundee.ac.uk/jpred/
PONDR-VSL2	Dunker et al., 2008^49^; Schiavina et al., 2021^50^	http://www.pondr.com/
APBS-PDB2PQR	Jurrus, et al., 2017^54^	https://server.poissonboltzmann.org/
HYDROPRO	Ortega et al., 2011^21^	N/A
ImageJ	Schneider et al., 2012^51^	https://imagej.nih.gov/ij/
Origin7.0	OriginLab	https://www.originlab.com/
PyMOL	DeLano Scientific LLC	http://www.pymol.org/

**Other**		

Expression and purification of wild-type protein	Mebus-Antunes et al., 2022^19^	https://journals.plos.org/plosone/article?id=10.1371/journal.pone.0264643
Expression and purification of R85C protein	This paper	N/A

### Resource availability

#### Lead contact

Further information and requests for resources, code and reagents should be directed to and will be fulfilled by the lead contact, Andrea T. Da Poian (dapoian@bioqmed.ufrj.br).

#### Materials availability

This study did not generate new unique reagents.

### Experimental model and subject details

#### Cultivation media and strains

*E. coli* strain BL21-DE3-pLysS (C606010, Thermo Fisher Scientific) was used in this study. Glycerol-stocks of the cells were kept at -80°C. The cells were cultivated in M9 minimal medium consisting of 200 mL base salt solution (240 mM Na_2_HPO_4_, 110 mM KH_2_PO_4_, 43 mM NaCl, pH 7.35); 2 mL 1M MgSO_4_ solution; 20 mL 20% glucose solution; 100 μL 1M CaCl_2_ solution; 1 mL thiamine solution (10 mg/mL), 40 mL NH_4_Cl solution (25 mg/mL), 1 mL chloramphenicol (34 mg/mL), and 1 mL ampicillin (34 mg/mL). The resulting solution was filled up to 1 L with sterilized water.

### Method details

#### Recombinant proteins

##### Expression and purification

The coding sequences of DENVC (serotype 2; residues 1-100) and its mutant R85C were cloned into pET3a by GenScript (Piscataway, NJ, EUA) and transformed into *E. coli* BL21-DE3-pLysS. For cultivation, the cells were streaked out on LB plates and incubated overnight at 37°C. Single colonies were picked and inoculated into a M9 preculture (20 mL in 100-ml flasks) and cultivated for 5 h to be subsequently diluted into a second preculture. The second preculture was used to inoculate the main culture (1L), which was cultivated at 37°C, at 200 rpm. Growth was monitored by measuring the optical density at 600 nm (OD_600_) and recombinant protein expression was induced with 0.5 mM isopropyl-D-1-thiogalactopyranoside (IPTG), overnight, at 30°C. The cells were then centrifuged (∼ 5,000 *g* for 30 min at 4°C) and the pellets were resuspended in lysis buffer consisting of 25 mM HEPES, pH 7.4, containing 0.2 M NaCl, 1 mM EDTA, glycerol 5% (v/v) and protease inhibitor cocktail (P8465, Sigma-Aldrich). The cells were disrupted by ultrasonication. After this step, the lysate was incubated with NaCl at a final concentration of 2 M and left on agitation for 60 min, at 4°C. The lysate was ultracentrifuged at 70,400 *g* for 50 min at 4°C. The supernatant was applied onto a HiTrap Heparin HP column and DENVC was purified using a step gradient with an increasing NaCl concentration (0.5 - 2 M). Fractions containing DENVC protein were confirmed by 18% SDS-PAGE gel, concentrated, and stored at – 20 °C. To prevent nonspecific disulfide bond formation during R85C expression, 1 mM DTT was added to all buffer solutions used.

##### Preparation of the covalent-linked dimer of R85C (R85C^ox^)

To generate a disulfide bond linking R85C subunits, the protein was incubated with 3 mM diamide at 4°C, with agitation, for 40 min, and applied in a HiTrap Heparin HP column coupled to an AKTA Start instrument (GE Healthcare). The protein was eluted from the column with a NaCl gradient (0.5 to 2 M) in 25 mM HEPES buffer (pH 7.4), containing 1 mM EDTA and 5% (v/v) glycerol, without DTT, with a flow of 5 ml/min. Purified R85C^ox^ was concentrated using an Amicon® centrifuge filter of 10 kDa cut-off (Merck-Millipore, USA) at 6,000×*g*, 4°C, in 55 mM NaH_2_PO_4_ buffer (pH 7.4), 200 mM NaCl, 2 mM PMSF, 5 mM EDTA, and 5 mM azide, and stored at −20°C. Protein concentrations were determined spectrophotometrically at 280 nm using the extinction coefficient of 11,000 M^–1^·cm^–1^.

##### Confirmation of disulfide bond formation

The formation of the disulfide bond covalently linking the mutant monomers (R85C^ox^) was confirmed by the observation of a ∼ 25 kDa band (dimer molecular mass) in a non-reducing 18% SDS-PAGE. Additionally, to quantify the remaining free sulfhydryl groups in the sample, 10 μM R85C^ox^ in 100 mM Tris-HCl buffer (pH 7.4), was incubated with 5,5'-dithiobis (2-nitrobenzoic acid) (DTNB) in a final concentration of 10 μM.^20^ The presence of free sulfhydryl groups was determined by measuring the absorbance at 412 nm using a BioMATE 3S UV-Vis spectrophotometer (Thermo Scientific, USA).

##### Preparation of the reduced (non-covalent dimer) R85C (R85C^red^)

To reduce R85C^ox^ disulfide bond, generating the mutant protein in the completed reduced form (R85C^red^), R85C^ox^ was incubated with 15 mM DTT for 40 min.

#### Dynamic light scattering (DLS)

DLS experiments were carried out using a Zetasizer Nano Series S90 (Malvern Instruments). Just before the experiments, DENVC WT, R85C^ox^, or R85C^red^, at 200 μM concentration in 55 mM NaH_2_PO_4_ buffer (pH 7.4) containing 200 mM NaCl, were centrifuged at 28,000×*g*, for 30 min, at 4°C, to remove any impurity in the sample. All the measurements were performed with the supernatant. The measurements were performed at 25°C, in a quartz cuvette with an optical path length of 10 mm. The results were represented as average values obtained from 30 scans by experiment. All experiments were carried out in triplicate. Additional experiments were performed using a ZetaPALS (Brookhaven Instruments Corp.) equipment. For these experiments, the protein concentration used was 10 μM.

#### Transmission electron microscopy (TEM)

R85C^ox^ and R85C^red^, at 5 nM concentration in 55 mM NaH_2_PO_4_ buffer (pH 7.4) containing 300 mM NaCl, and 5 mM EDTA, were kept for 1 hour at room temperature to allow NCLPs formation. Alternatively, R85C^ox^ was incubated with 15 mM DTT after being maintained in the condition for NCLP assembly (1 h at room temperature at 5 nM concentration). Then 3.5 μL of the sample was applied on carbon-coated 400 mesh copper grids (Electron Microscopy Sciences), previously subjected to glow discharge in a Pelco Easiglow™ (15 mA, 0.39 mbar, for 25 sec). After 10 min, samples were washed three times with 3.5 μL of distilled and filtered MilliQ water, the excess liquid was gently dried with filter paper, and the grids were stained with 0.5% sodium phosphotungstate (PTA) for 30 sec. Images were acquired using a FEI Tecnai Spirit microscope operated at 120 kV coupled to a ‘2k×2k’ pixel Veleta C.C.D. Camera (Olympus). Image magnification ranged from 68,000 to 150,000 times. Particles’ counting and their Feret’s diameter measurement were performed using ImageJ software.^51^

#### Thermal denaturation studies

##### Circular dichroism (CD) analysis

CD spectroscopy experiments were performed in JASCO J815 (Tokyo, Japan) equipped with a Peltier type temperature controller using 0.1 cm path length quartz cuvette with DENVC WT, R85C^ox,^ and R85C^red^ proteins at 13 μM concentration in buffer 55 mM NaH_2_PO_4_ (pH 7.4) containing 200 mM NaCl. Spectra were acquired between 200 and 310 nm at different temperatures, with data pitch of 0.2 nm, bandwidth of 2 nm, scanning speed of 100 nm/min with a data integration time of 4 seconds and performing 3 accumulations. For thermal denaturation/renaturation curves, ellipticity was acquired at 222 nm, in intervals of 1°C from 25°C to 95°C, and then from 95°C to 25°C. Results were expressed as molar ellipticity [Θ] (deg.cm^2^.dmol^-1^), after buffer and baseline subtractions, according to:

$${\left[\Theta \right]}_{\lambda }=\frac{\theta }{c*l*10*n}$$

where θ being the measured ellipticity at wavelength λ (deg), c is the protein concentration (mol/L), l the path length of the cuvette (in cm), and n the number of amino acid residues in the protein.

##### Differential scanning calorimetry (DSC)

DSC experiments were performed using an N-DSC III apparatus (TA Instruments, USA) in the range from 20 to 95°C with scan rates of 1.0°C/min. Initially, both calorimeter cells were loaded with the buffer solution, equilibrated at 20°C for 10 min, and scanned repeatedly until the baseline was reproducible and stable. Then, the sample cell was loaded with the DENVC WT, R85C^ox^ or R85C^red^ at final concentrations of 42.5 μM in 55 mM NaH_2_PO_4_ buffer (pH 7.4) containing 200 mM NaCl. The baseline correction was obtained by subtracting the buffer scan from the protein scan using the software Launch NanoAnalyze, supplied by TA Instruments. The thermograms were fitted using a two-state model with the program Origin 7.0. The Van’t Hoff equation was used to calculate the ${\Delta H}_{VH}$ involved in the transition:

$$\frac{d}{d_{\frac{1}{T}}}\ln\left(K_{u}\right)=-\frac{\Delta H}{R}$$

where ${\Delta H}_{VH}$ is the enthalpy change, R is the universal gas constant, and $K_{u}$ is the denaturation constant at the correspondent temperature, T.

Calorimetric enthalpy change (${\Delta H}_{cal})$ was calculated from the thermogram area. Gaussian fits and the deconvolution analysis were performed using the program Origin 7.0 by adjusting a minimum number of Gaussian curves to the DSC thermograms, and Gaussian curves were treated as described above.

#### Chemical denaturation studies

##### Fluorescence spectroscopy

The effects of guanidine hydrochloride (Gd:HCl) (Sigma-Aldrich) on the structures of DENVC WT and R85C proteins were measured by the intrinsic fluorescence of the single tryptophan residue of DENVC (W69), using a Cary Eclipse (Agilent Technologies) fluorescence spectrophotometer equipped with a quartz cuvette of 1 cm path length and excitation and emission slits of 5 nm. The assays were performed at 25°C. The protein samples at 5 μM concentration in 55 mM NaH_2_PO_4_ buffer (pH 7.4) containing 200 mM NaCl, were incubated for 5 min with from 0 to 7.4 M Gd:HCl before the fluorescence analysis. The intrinsic fluorescence was measured by exciting the samples at 280 nm and collecting the emission spectrum from 290 to 450 nm. Each measurement in the emission spectrum was an average of 10 scans. The data were corrected by subtracting the fluorescence values obtained for a buffer solution with the respective Gd:HCl concentration. The center of spectral mass (CM) of the emission spectrum was quantified as follows:

$$CM=\frac{\sum \left(v_{i}\;x\;F_{i}\right)}{\sum F_{i}}$$

where $F_{i}$ is the fluorescence emission at wavelength $v_{i}$, and the summation is performing over the range of measured values of F.

#### Secondary structure and order/disorder prediction

Secondary structure prediction was performed using JPRED4 server (https://www.compbio.dundee.ac.uk/jpred/). JPRED4/JNET assigns a confidence score varying from 0 (low) to 9 (high). JNET uses a neural network that includes learning and training algorithms based on the increasing number of known protein secondary structures. The confidence level of a given prediction gives information on the tendency of a given residue to be in a secondary structure.^48^

Order/disorder content in protein structure was predicted using PONDR server (http://www.pondr.com/). PONDR uses neural networks than can be trained using different inputs. VSL2 is an PONDR predictor trained by combining two predictors, for long sequences (> 40 residues) and short 8 to 9 residues sequences,^49^ using not only crystallographic structures but also NMR structures and circular dichroism secondary structure data. VSL2 showed the best match to experimentally determined structural and dynamical features of DENVC.^17^^,^^52^^,^^53^

### Quantification and statistical analysis

Quantification, data analysis, and plotted graphs were performed using Origin 7.0. For transmission electron microscopy images, n = 598 is the number of particles analyzed for R85C^ox^ and, n = 177 is the number of particles analyzed for R85C^red^. The mean Feret’s diameters values were used to calculate the standard deviation (SD) between the diameter measurements.

The electrostatic potential values were calculated in APBS software with the protonation states and charge values determined by the PDB2PQR server along with PROPKA program (pH 7.0, 200 mM NaCl, 25°C).^54^

## Data Availability

• Original SDS-PAGE images have been deposited at Mendeley Data and are publicly available as of the date of publication. The access number is listed in the key resources table. • Raw data derived from measurements of dynamic light scattering, transmission electron microscopy, thermal and chemical denaturation studies have been deposited at Mendeley Data and the access number are listed in the key resources table. • All original codes have been deposited at Mendeley Data and are publicly available as of the date of publication. DOI are listed in the key resources table. • Any additional information required to reanalyze the data reported in this paper is available from the lead contact upon request.
