# Supplementary material for: Self-assembly of dengue virus empty capsid-like particles in solution
Source: iScience. 2023 Feb 14;26(3):106197. doi: 10.1016/j.isci.2023.106197 (PMC9986514; doi:10.1016/j.isci.2023.106197)
Supplement: Document S1. Figures S1–S4 [file mmc1.pdf]

iScience, Volume *26*

Supplemental information

Self-assembly of dengue virus empty capsid-like particles in solution

Thais C. Neves-Martins, Nathane C. Mebus-Antunes, Carlos H.G. Neto, Glauce M. Barbosa, Fabio C.L. Almeida, Icaro P. Caruso, and Andrea T. Da Poian

SUPPLEMENTAL MATERIAL

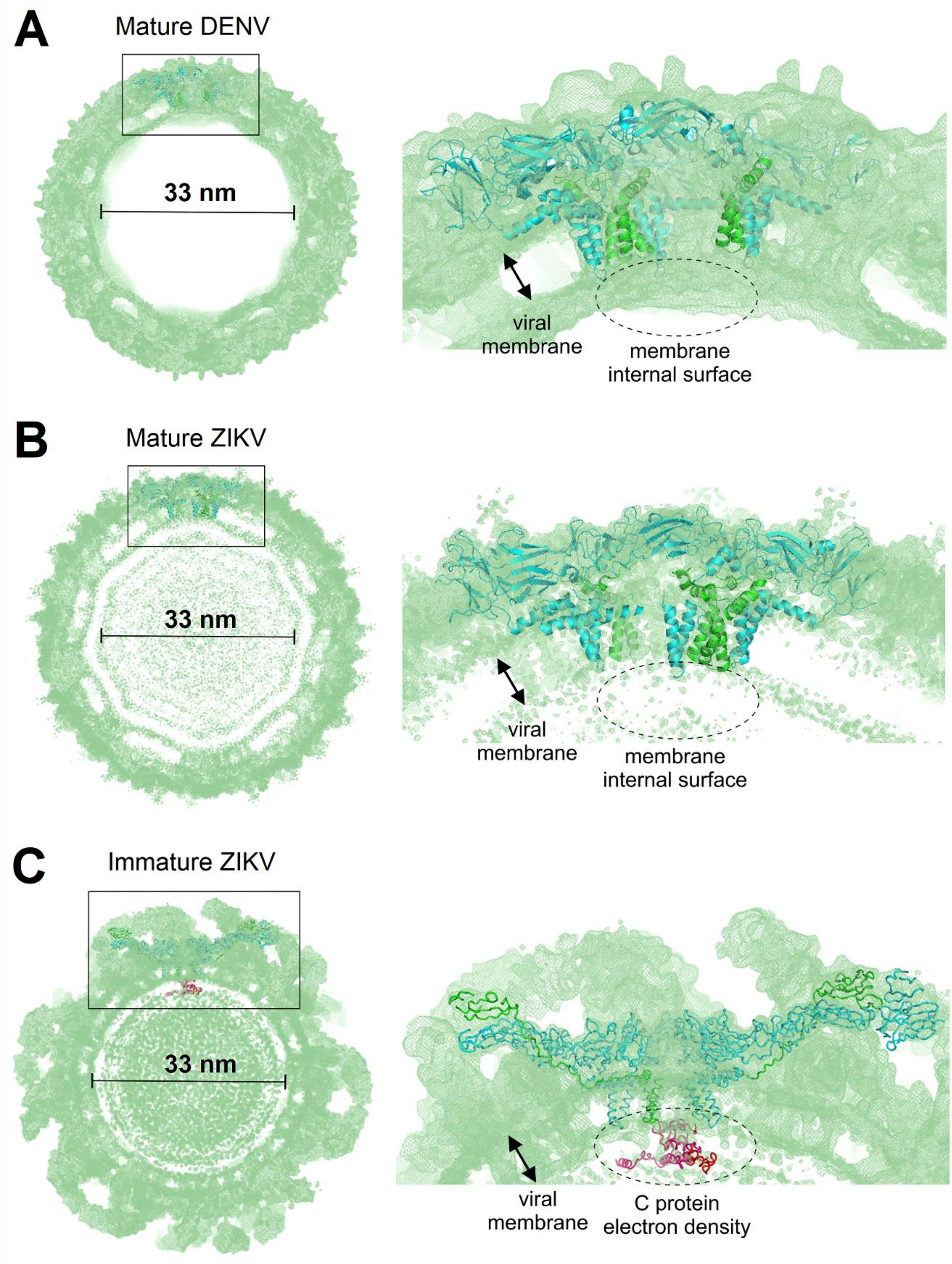

Figure S1. Structure of DENV and ZIKV virions (Related to Figure 3). Cross section of mature DENV (A) and ZIKV (B), and immature ZIKV (C) particles showing the radial cryo-EM density distribution, with the ribbon structures of the E (blue) and M (green) proteins superimposed to the density maps. In the immature virus (C) 60 trimeric spikes composed of E (blue) and the precursor M (prM, green) proteins form the outer icosahedral lattice. The proposed putative orientation of C protein (red) is also represented. The figures were prepared using cryo-EM structures deposited in PDB (mature DENV: 3J27; mature ZIKV: 5IRE; immature ZIKV: 6LNT).

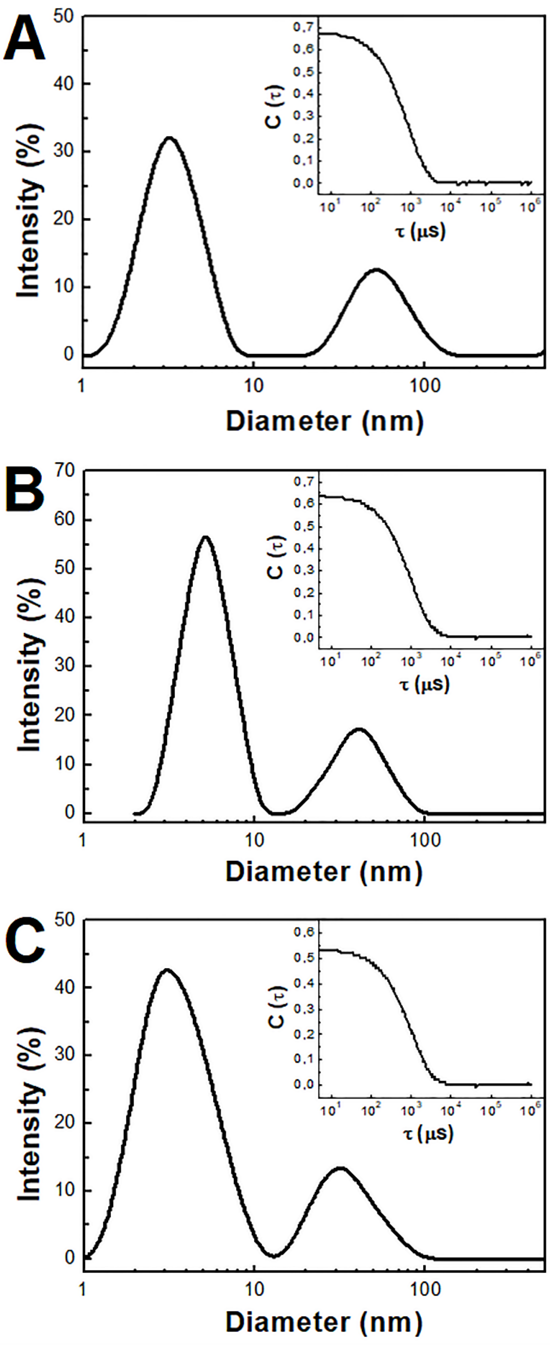

Figure S2. Hydrodynamic diameter distribution of DENVC R85C mutant in different steps of protein purification (Related to Figure 3). Size distribution profiles of (A) R85C sample eluted from the first step affinity chromatography in the elution buffer (HEPES 25 mM, EDTA 1 mM, glycerol 5% (v/v), pH 7.4), containing 1.5 M NaCl and 1 mM DTT; (B) R85C protein after incubation with diamide, eluted from the second step affinity chromatography in the elution buffer (HEPES 25 mM, EDTA 1 mM, glycerol 5% (v/v), pH 7.4), containing 1.5 M NaCl, without DTT; and (C) R85C protein after buffer exchange for 55 mM NaH_2PO_4, 200 mM NaCl, pH 7.4. The insets show the respective correlation curves. DLS measurements were performed in a ZetaPALS (Brookhaven Instruments Corp.) equipment, using proteins at a 10 μM concentration. The results are represented as average values from 10 scans by experiment.

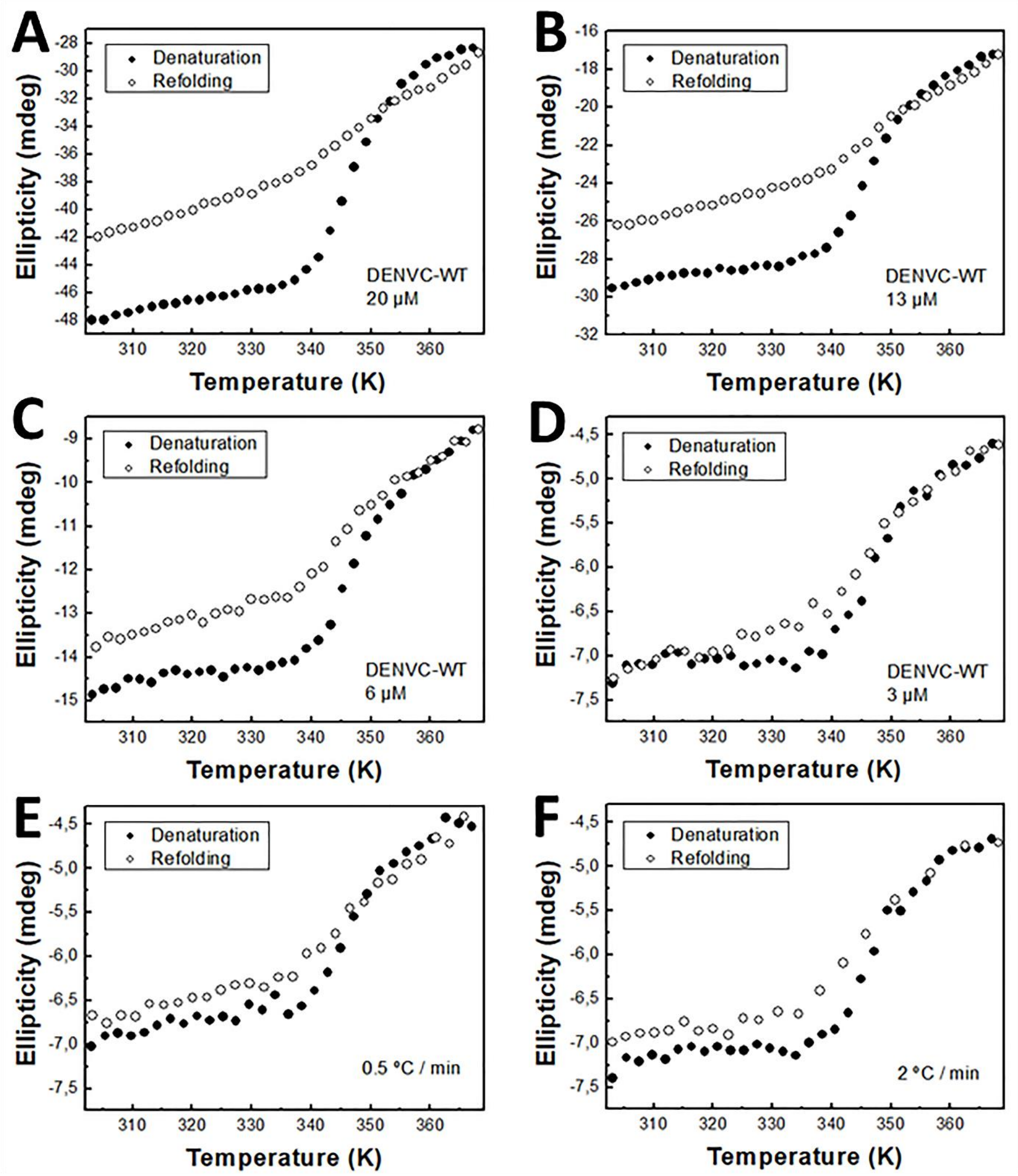

Figure S3. CD analysis of the thermal dissociation/denaturation process of DENVC WT at different concentrations (Related to Figure 5). Thermal dissociation/denaturation by collecting the ellipticity at 222 nm from 298 to 368 K (filled circles) and return (empty circles) for DENVC-WT at 20 μM (A), 13 μM (B), 6 μM (C), and 3 μM (D) concentrations. The data were obtained with a scan rate of 1.0°C/min. At 3 μM concentration, thermal dissociation/denaturation process was also analyzed using 0.5°C/min (E) or 2°C/min (F). The samples were prepared in 55 mM NaH_2PO_4 buffer (pH 7,4) with 200 mM NaCl, and all experiments were performed at least twice.

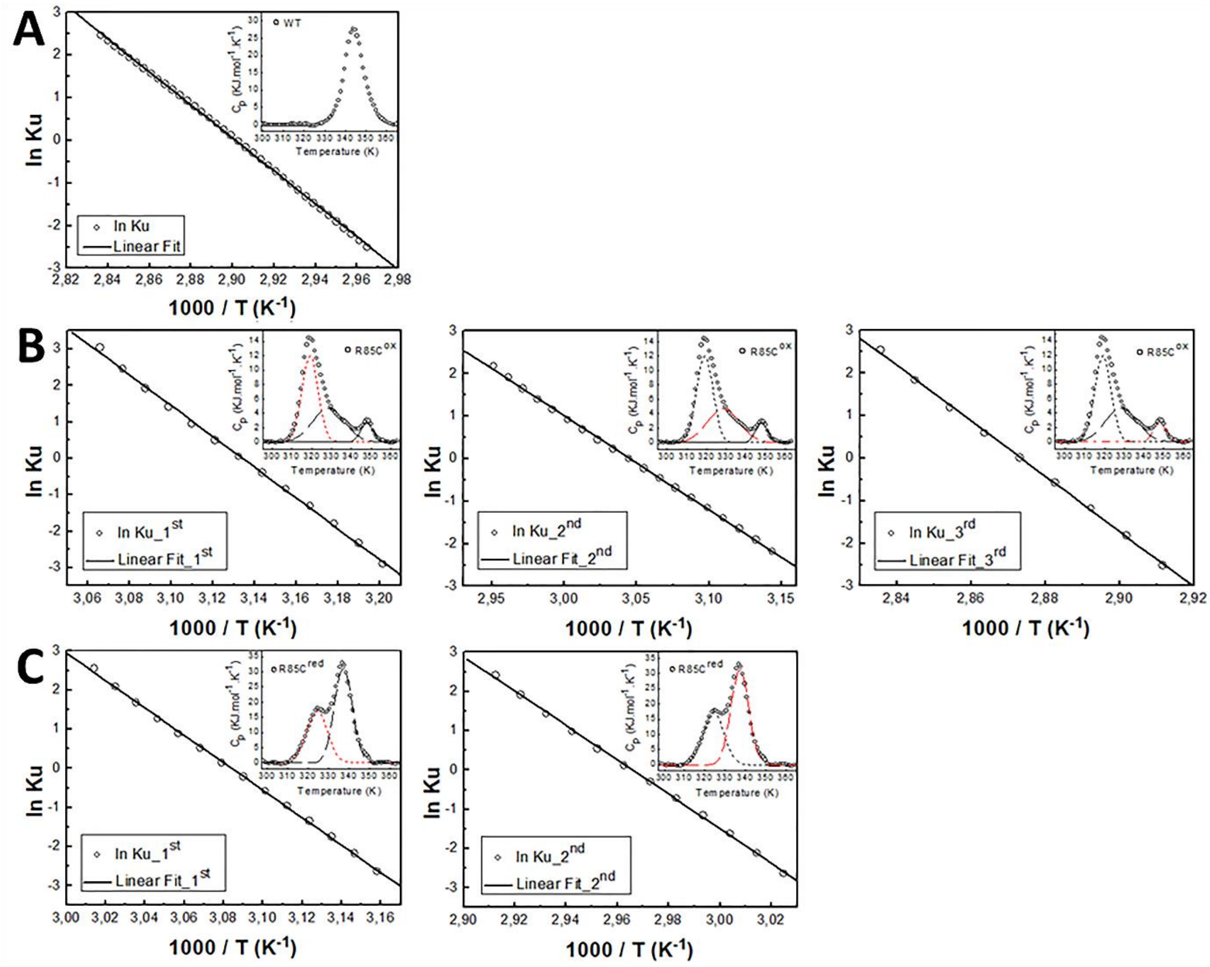

Figure S4. Thermogram treatment for Van't Hoff enthalpy calculation (Related to Figure 6). The thermogram treatment was performed using multiple Gaussian adjustments to the experimental data (See Material and Methods). ΔH_{VH} (Van't Hoff enthalpy variation) was obtained for DENVC-WT (A), R85C^{ox} (B), and R85C^{red} (C). The inserts show the thermogram for all proteins and the Gaussian fit for the R85C^{ox} (B), and R85C^{red} (C) are represented by red dashed lines. Measurements were performed from 293 to 368 K with a scan rate of 1°C/min. All samples were in buffer 55 mM NaH_2PO_4 (pH 7.4) with 200 mM NaCl. Measurements were performed at least twice, and fittings were obtained using Origin 7.0.
